# Medullary cavity application of tranexamic acid to reduce blood loss in tibial intramedullary nailing procedures—a randomized controlled trial

**DOI:** 10.1007/s00264-023-05824-8

**Published:** 2023-05-04

**Authors:** Cong Xiao, Zhixiang Gao, Wei Yu, Kai Yao, Yang Cao, Nengji Long, Shaoyun Zhang, Yishan Jiang

**Affiliations:** Department of Orthopedics, The Third Hospital of Mianyang, Sichuan Mental Health Center, No. 190 The East Jiannan Road, 621000 Mianyang, China

**Keywords:** Tranexamic acid, Tibial fractures, Intramedullary nails, Hidden blood loss

## Abstract

**Purpose:**

Studies have shown an average postoperative hidden blood loss (HBL) of 473.29 ml and an average Hb loss of 16.71 g/l after intramedullary nailing. Reducing HBL has become a primary consideration for orthopaedic surgeons.

**Methods:**

Patients with only tibial stem fractures who visited the study clinic between December 2019 and February 2022 were randomized into two groups using a computer-generated form. Two grams of tranexamic acid (TXA) (20 ml) or 20 ml of saline was injected into the medullary cavity before implantation of the intramedullary nail. On the morning of the surgery, as well as on days one, three and five after surgery, routine blood tests and analyses of CRP and interleukin-6 were completed. The primary outcomes were total blood loss (TBL), HBL, and blood transfusion, in which the TBL and HBL were calculated according to the Gross equation and the Nadler equation. Three months after surgery, the incidence of wound complications and thrombotic events, including deep vein thrombosis and pulmonary embolism, was recorded.

**Results:**

Ninety-seven patients (47 in the TXA group and 50 in the NS group) were analyzed; the TBL (252.10 ± 10.05 ml) and HBL (202.67 ± 11.86 ml) in the TXA group were significantly lower than the TBL (417.03 ± 14.60 ml) and HBL (373.85 ± 23.70 ml) in the NS group (*p* < 0.05). At the three month postoperative follow-up, two patients (4.25%) in the TXA group and three patients (6.00%) in the NS group developed deep vein thrombosis, with no significant difference in the incidence of thrombotic complications (*p* = 0.944). No postoperative deaths or wound complications occurred in either group.

**Conclusions:**

The combination of intravenous and topical TXA reduces blood loss after intramedullary nailing of tibial fractures without increasing the incidence of thrombotic events.

## Introduction

In adults, approximately 2% of all fractures are tibial shaft fractures. The most common cause of these fractures is high-energy trauma (traffic trauma, fall from a height, crushing injury, etc.) [1]. There are several ways to treat tibial shaft fractures, including conservative treatment and orthopaedic surgery using intramedullary nailing, minimally invasive percutaneous plate osteosynthesis (MIPPO), and external fixation (Ilizarov frame and hybrid fixators) [2–4]. Fixation with intramedullary nails acts as a three-point intramedullary fixation, providing sufficient stability to allow early weight-bearing while walking to reduce soft tissue complications, thus becoming the standard of treatment for tibial stem fractures [5, 6]. According to the relevant data, the amount of intraoperative bleeding related to tibial intramedullary nailing, regardless of the suprapatellar or infrapatellar approach, is very small, approximately 20 ml. Nevertheless, only explicit bleeding is reported, and hidden blood loss (HBL) is not included [7]. In 1966, Nelson and Brown [8] proposed that HBL was a complication of postoperative surgery. Wang et al. [9] observed that large amounts of HBL may occur after tibial intramedullary nailing, with a mean volume of approximately 473.29 ± 102.75 ml and a mean Hb loss of 16.71 ± 4.79 g/l. The postoperative transfusion rate increases from 2.25% for plate fixation to 5.96% for intramedullary nailing [10]. Some patients, however, require allogenic blood transfusions during the perioperative period because of blood loss. Additionally, transfusions carry a high cost and are associated with side effects, infectious diseases, and immune system inhibition [11]. Consequently, minimizing blood loss and blood transfusion related to orthopedic surgery remains a prior concern for clinicians during the perioperative period.

Tranexamic acid (TXA), which is a synthetic antifibrinolytic agent that inhibits the production of plasminogen by competitively binding to its lysine-binding sites to enhance the effectiveness of the patient’s own haemostatic mechanisms, has been used for many years to reduce blood loss in orthopaedic surgery [12]. It was demonstrated that the combination of intravenous and topical TXA may be more effective than topical or intravenous (IV) TXA alone in hip and knee arthroplasty [13, 14]. However, there is limited literature on the combined intravenous and topical application of TXA in the perioperative period in traumatic orthopaedic patients, with only three randomized controlled studies showing that the combined application significantly reduces perioperative bleeding and transfusion rates [15]. Therefore, we conducted a randomized controlled trial to evaluate the efficacy and safety of the combined application of intravenous and topical TXA in patients undergoing intramedullary nailing of the tibia. We hypothesized that the combination of an intravenous injection and topical TXA may be more effective than intravenous TXA alone in reducing blood loss without increasing the risk of associated complications such as thrombotic events.

## Materials and methods

**Fig. 1:**
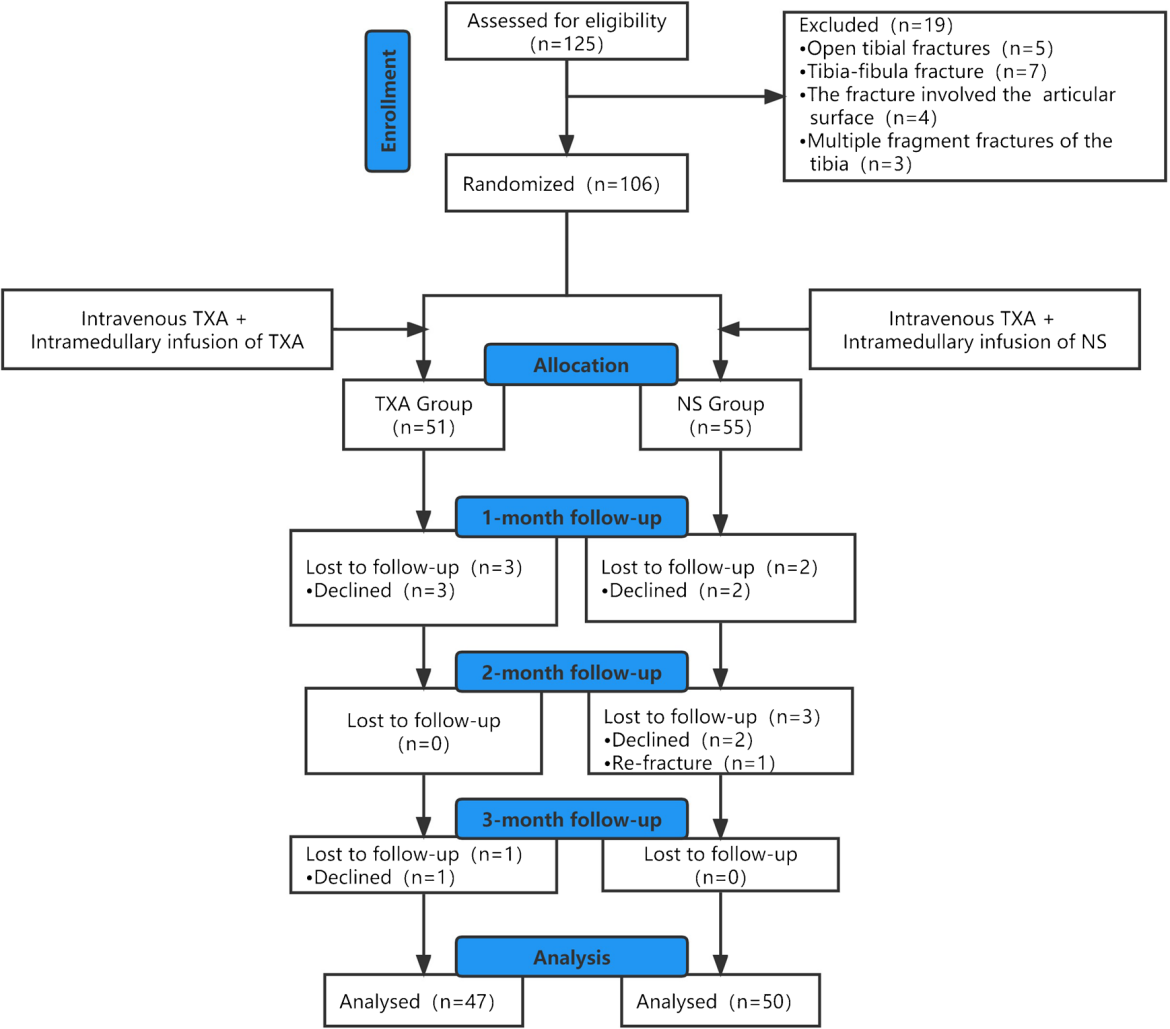
Patient flow chart

Ethics approval was obtained from the hospital’s ethics committee for this study, and the approval was registered on the Chinese Clinical Trial Registry platform (No. ChiCTR2100051947). The study was performed in accordance with the Declaration of Helsinki and good clinical practice guidelines [16]. Ninety-seven patients presenting with unilateral tibial stem fractures between December 2019 and February 2022 were recruited for this study, and informed written consent was obtained from all patients (Fig. 1).

### Inclusion and exclusion criteria

Patients aged > 18 years with a diagnosis of unilateral tibial fracture were included. Patients aged < 18 years with open fracture; pathological fracture or old tibial fracture; allergy to TXA; preoperative blood transfusion; history of pulmonary embolism (PE) or deep vein thrombosis (DVT); abnormal liver, kidney, or coagulation function; and incomplete follow-up data were excluded. In this study, two doctors classified fractures in accordance with the AO/OTA classification. The patients were randomly divided into two groups with the help of a computer-generated table (www.randomizer.org). During the study period, only the randomized study personnel had access to the random number table, and the surgeons and nurses prepared the solutions while knowing the TXA or NS assignments. The anesthesiologist, patient, and postoperative data collector were unaware of the assignments.

### Perioperative management

All procedures were performed by the same experienced surgeon, and the anesthesiologist decided whether to use general or spinal anaesthesia without the use of tourniquets. In all patients, the implant used was the infrapatellar approach intramedullary nailing system (Xiamen Dabo) provided by the same manufacturer.

None of the patients included in the study used TXA from the time of hospitalization until the day before surgery. For Intravenous, the effective concentration of tranexamic acid typically falls between 10 and 20 mg/ml. A loading dose is typically administered before, or at the start of, surgery, followed by maintenance doses. For localized use, such as in the case of tranexamic acid use for hemostasis during orthopaedic surgery, the concentration can be as high as 50–100 mg/ml [17]. This allows for 2 g of tranexamic acid to be diluted in 20–40 ml for topical application. TXA group: One gram of TXA was injected intravenously 15 min before skin incision, and 2 g of TXA (NS diluted to 20 ml) was injected into the medullary cavity before intramedullary nail implantation. NS group: One gram of TXA was injected intravenously 15 min before skin incision, and 20 ml of saline was injected into the medullary cavity before intramedullary nail implantation. The end caps of the intramedullary nails were implanted, and the incisions were closed in layers.

Blood transfusions were performed according to the guidelines for perioperative blood transfusion management issued by the Chinese Ministry of Health. A blood transfusion was recommended according to the following guidelines: (1) hemoglobin concentration < 70 g/l and (2) haemoglobin concentration < 80 g/l, poor tolerance to anaemia, or anaemia-related symptoms, such as dizziness, fever, palpitations, and shortness of breath.

### Extraction of data, outcome measuring, and quality assessment

On the morning of surgery and on postoperative days one, three and five, routine blood tests and assessments of CRP, D-dimer, and interleukin-6 were performed. The researchers involved in data collection were blinded to the patients’ intervention plans.

There were four primary outcomes: total blood loss (TBL), HBL, intraoperative blood loss (IBL), and blood transfusion, in which the TBL and HBL were calculated according to the Gross equation [18] and the Nadler equation [19]. The secondary outcomes included wound complications; CRP, interleukin-6, and D-dimer levels; the incidence of thrombotic events, including deep vein thrombosis and pulmonary embolism; and mortality within three months after surgery. At postoperative day one, two weeks, one month, two months, and three months, follow-up ultrasounds of the lower extremity veins were performed.

### Statistical analysis

In this study, chi-square tests were conducted on categorical variables expressed as percentages, and Student’s *t*-test was used to compare differences between groups based on continuous variables with standard deviations. Patient survival within three months after surgery was analyzed using the Kaplanas used to compare diff We considered *p* values of less than 0.05 to be statistically significant. Statistical analyses were conducted using SPSS 23.0.

## Results

Over the course of the study, 97 patients met the inclusion criteria. The numbers of participants in the TXA and NS intervention groups were 47 and 50, respectively. Statistically, there were no significant differences between the two groups based on their baseline data (*p* > 0.05) (Table 1).

**Table 1:** Baseline demographic characteristics

	TXA group (*n* = 47)	NS group (*n* = 50)	*t*/*χ*^2^	*p*
Age (year)	42.40 ± 9.93	39.64 ± 9.16	1.426	0.157
Sex, *n* (%)				
Male	22 (46.81)	30 (60.00)	1.695	0.193
Female	30 (53.19)	20 (40.00)		
Weight (kg)	62.34 ± 11.98	64.37 ± 15.95	0.705	0.483
Height (m)	1.69 ± 0.07	1.71 ± 0.03	1.739	0.085
BMI (kg/m^2^)	21.84 ± 4.23	22.04 ± 5.52	0.198	0.843
ASA, *n* (%)				
I	13 (27.66)	12 (24.00)	0.169	0.919
II	26 (55.32)	29 (58.00)		
III	8 (17.02)	9 (18.00)		
AO/OTA classification				
A	19 (40.43)	21 (42)	1.174	0.56
B	21 (44.68)	25 (50)		
C	7 (14.89)	4 (8)		
Anesthesia type, *n* (%)				
Spinal	9 (19.15)	12 (24.00)	0.336	0.562
General	38 (80.85)	38 (76.00)		
Surgical duration (min)	80.49 ± 10.09	77.64 ± 12.61	1.224	0.224
IMN diameter (cm)	9.85 ± 0.55	9.88 ± 0.33	0.316	0.753

*ASA*, American Society of Anesthesiologists; *IMN*, intramedullary nailing

Perioperative TBL was lower in the TXA group (252.10 ± 10.05 ml) than in the NS group (417.03 ± 14.60 ml), and the differences were statistically significant between the two groups (*p* = 0.000). However, no statistically significant differences in IBL were found between the two groups (29.27 ± 1.46 ml vs. 29.47 ± 2.09 ml, *p* = 0.594). On postoperative day 3, the HBL (calculated according to the Hct) in the NS group was significantly higher than that in the TXA group (373.85 ± 23.70 vs. 202.67 ± 11.86, *p* = 0.000) (Table 2). Two patients (4.26%) in the TXA group and three patients (6.00%) in the NS group received a blood transfusion. In both groups, there were no statistically significant differences (*p* = 0.943) (Table 2).

**Table 2:** Perioperative blood loss

	TXA group (*n* = 47)	NS group (*n* = 50)	*t*/*χ*^2^	*p*
TBL (mean ± SD, ml)	252.10 ± 10.05	417.03 ± 14.60	64.409	0.000
HBL (mean ± SD, ml)	202.67 ± 11.86	373.85 ± 23.70	44.539	0.000
IBL (mean ± SD, ml)	29.27 ± 1.46	29.47 ± 2.09	0.535	0.594
Blood transfusion, *n* (%)	2 (4.26)	3 (6.00)	0.005	0.943

*TBL*, total blood loss; *IBL*, intra-operative blood loss; *HBL*, hidden blood loss

In the preoperative CRP (7.47ts (4.26%) in the T mg/l) and IL-6 (7.77 ± 0.98 vs. 8.05 ± 0.72 pg/ml) comparisons between the TXA and NS groups, there were no statistically significant differences (*p* > 0.05).

**Fig. 2:**
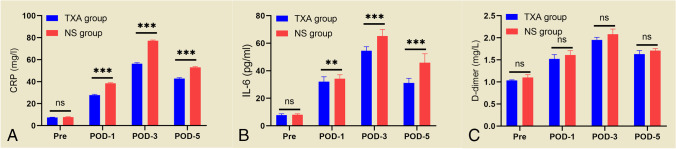
Changes in CRP (**A**), IL-6 (**B**), and D-dimer (**C**) on preoperative and postoperative days 1, 3, and 5 in both groups

**Fig. 3:**
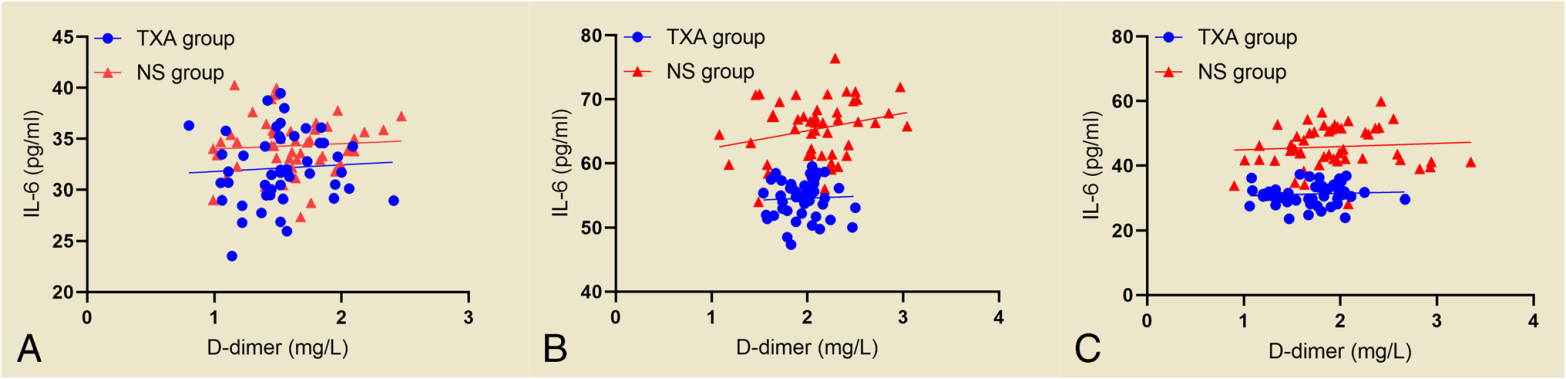
Changes in the correlation between IL-6 and D-dimer on postoperative days 1 (**A**), 3 (**B**), and 5 (**C**) in both groups

The serum levels of CRP (27.86XA and NS groups, there w) and IL-6 (32.14 ± 3.49 vs. 34.32 ± 2.75 pg/ml) in both groups were higher on the first postoperative day, and the highest levels of CRP (56.40 ± 3.05 vs. 77.22 ± 2.42 mg/l) and IL-6 (54.59 ± 2.90 vs. 65.30 ± 4.70 pg/ml) were obtained on the third postoperative day. There was a gradual decrease in CRP (42.80 ± 2.80 vs. 53.07 ± 2.12 mg/l) and IL-6 (31.18 ± 3.31 vs. 45.85 ± 6.52 pg/ml) on postoperative day five, with a statistically significant difference between the two groups (*p* < 0.05) (Fig. 2A, B). In both groups, the serum postoperative D-dimer levels were significantly higher than the preoperative levels, but there was no statistically significant difference in the serum D-dimer levels between the two groups on postoperative days one, three and five (*p* > 0.05) (Fig. 2C). Correlation analysis revealed a positive correlation between serum IL-6 levels and D-dimer levels in the patients on postoperative days one, three and five in both the TXA and NS groups, but the correlation coefficient was low (Fig. 3).

At the 3-month postoperative follow-up, there were two patients (4.25%) in the TXA group and three patients (6.00%) in the NS group with deep vein thrombosis, with no significant difference in the incidence of thrombotic complications (*p* = 0.944, Table 3). No postoperative deaths or wound complications occurred in either group.

**Table 3:** Complications at postoperative 3 months

	TXA group (*n* = 47)	NS group (*n* = 50)	*χ*^2^	*p*
Thrombotic events, *n* (%)	2 (4.25)	3 (6.00)	0.005	0.944
Deep vein thrombosis	2	3		
Pulmonary embolism	0	0		
Wound complications, *n* (%)	0	0		
3 months mortality, *n* (%)	0	0		

## Discussion

This study attempted to compare the safety and efficacy of 2 g TXA by intramedullary infusion in patients with tibial intramedullary nailing during the perioperative period; topical use of 2 g TXA was found to be effective in reducing TBL and HBL and reducing inflammatory biomarkers, including CRP and IL-6. Our study showed no differences in the transfusion rates between the TXA and NS groups; we also found no differences in thrombotic events and mortality risk at three months postoperatively in the two groups.

TXA is a synthetic lysine derivative with a molecular weight of 157.2 g/mol that competitively occupies the lysine binding site of fibrinogen and thus blocks the lysine binding site on the plasminogen molecule to achieve haemostasis [20]. In 1986, after TXA was demonstrated to effectively control bleeding in haemophiliac patients during dental surgery [21], this agent was also extensively studied to reduce bleeding in extracorporeal circulation and orthopaedic surgery, including spine [22], knee, and hip replacement surgeries [23, 24]. In several studies, researchers reported that intravenous TXA alone, topical TXA alone, and intravenous TXA combined with topical TXA effectively reduce TBL and HBL without increasing the complications of thrombosis, but intravenous TXA combined with topical TXA is more effective [13, 14]. However, the literature on the use of TXA in traumatic orthopaedic cases is limited. Therefore, TXA was applied topically and intravenously in this study to assess the effect on perioperative blood loss and inflammatory biomarkers.

The blood supply in the bone marrow cavity is maintained by arteriovenous trophoblastic pores, and there is a large network of bone marrow capillaries, i.e., blood sinuses. The bone marrow cavity has one to two large venous sinuses that receive blood from the transversely distributed venous ducts, which are connected to the systemic venous return system through the central venous sinus [25, 26]. The increase in HBL after tibial intramedullary nailing is mainly considered to be due to the opening of the intramedullary sinuses during the process of medullary reaming, with a large amount of blood retention in the medullary cavity and leakage through the fracture into the surrounding muscle tissue space. In our study, the amount of HBL in the NS group was 373.85 ± 23.70 ml lower than that in the study by Wang et al. [9], who reported that the amount of HBL can reach 473.29 ± 102.75 ml, which was related to the administration of intravenous TXA before incision of skin in our study.

The intravenous administration of TXA reduces the need for transfusion and the amount of perioperative blood loss, and when applied intravenously in high doses, TXA is widely distributed extracellularly and intracellularly. It spreads rapidly to local bleeding sites and has an approximate three h biological half-life [27]. However, there is a dose–effect relationship for TXA, and there is also a potential risk of thromboembolism with high doses of TXA. Topical TXA increases the local drug concentration, thus avoiding the blockage of fibrin degradation at other sites caused by intravenous administration, and it has the following advantages: (1) direct action at the bleeding site and a high drug concentration and (2) prevention of systemic side effects caused by a high intravenous TXA concentration. Applying 1 g or 3 g TXA topically after a hip fracture reduces blood loss in the early postoperative period without increasing the risk of acute coronary syndrome (ACS), venous thromboembolism (VTE), cerebrovascular accident (CVA), surgical site infection (SSI), or 90-day mortality in patients [15]. Despite the effectiveness of intravenous TXA in reducing perioperative blood loss, Wang et al. [28] reported that topical TXA has a high safety profile. In our study, the amounts of HBL (202.67 ± 11.86 ml) and TBL (252.10 ± 10.05 ml) in the TXA group were lower than those in the NS group, in which the HBL and TBL were 373.85 ± 23.70 ml and 417.03 ± 14.60 ml, respectively. In fact, only a difference of approximately 170 ml was found between the two groups of the study. Although significant, this is probably not a clinically meaningful difference. The HBL is less than that of femoral intramedullary nailing, which may be related to the small diameter of the tibial medullary cavity and the small sample size; further validation with a larger sample size is needed in the future. The combination of the intravenous and intramedullary application of TXA achieves high local concentrations and allows TXA to enter the system through the intramedullary bone marrow capillary network, further increasing the systemic TXA concentration. Despite this high concentration of TXA, the incidence of postoperative thrombotic events was not increased. Two patients in the TXA group (4.25%) and three in the NS group (6.00%) had intermuscular venous thrombosis within three months after surgery in both groups, with no significant difference in the incidence of thrombotic complications.

Trauma and surgical stress responses are associated with significantly elevated numbers of suppressive CD14 + HLA-DR^low^/-monocytes and CD16^BRIGHT^ CD62L^DIM^ neutrophils, leading to activation of inflammatory factors such as interleukin 6 and inducing a systemic inflammatory response syndrome [29]. TXA has been shown to affect the inflammatory response by reducing C5a production during fibrinolysis mediated by fibrinogen activator (tPA) and by confounding chymotrypsin-like protease activity [30]. Several clinical studies, including those involving cardiopulmonary bypass (CPB) [31], total hip arthroplasty [32], and total knee arthroplasty [33] patients treated with TXA during the perioperative period, have reported significantly reduced postoperative IL-6 levels in such cases. In our study, the CRP and IL-6 levels were lower after combined intravenous and local application of TXA than after intravenous TXA alone; however, unfortunately, we did not set up a negative control group for further comparison. In future studies, these parameters should be added to help interpret and compare the results. D-dimer in serum induces the synthesis and release of biologically active IL-1β and IL-6 from monocytes; therefore, serum IL-6 varies with D-dimer levels [34]. We found that the level of IL-6 in serum was positively correlated with the level of D-dimer in both the TXA and NS groups of patients by correlation analysis. It has been shown that inflammatory factors such as IL-6 are highly correlated with thrombosis, and therefore, anti-inflammatory activity in the vascular system may be another strategy to prevent or control thrombosis [35].

In conclusion, intravenous combined with topical TXA can achieve higher therapeutic concentrations directly at the site of bleeding, while systemic antifibrinolysis minimizes bleeding without increasing TXA-related adverse events. The following limitations were identified in the study:

1. The sample size was small, so the inclusion of a larger number of cases in the future will allow more credible results.
2. A negative control group and information on the local application of TXA alone were not provided, and there were no comparisons of whether local intramedullary infusion of TXA could achieve the effects of intravenous TXA alone or the effects of intravenous combined with local application of TXA. Controlled trials should be performed in the future.
3. Two to three grams of TXA were injected subfascially and intramuscularly in patients with hip fractures and femur fractures before closing the incision [36–39], but the dose of TXA in this study was only 2 g. In future studies, researchers will investigate the local application of different doses of TXA.

## Data Availability

Upon request, data and materials can be made available.

## Code availability

No code availability at this time.
